# Bipolar disorder and antithyroid antibodies: review and case series

**DOI:** 10.1186/s40345-016-0046-4

**Published:** 2016-02-11

**Authors:** Alberto Bocchetta, Francesco Traccis, Enrica Mosca, Alessandra Serra, Giorgio Tamburini, Andrea Loviselli

**Affiliations:** Unit of Clinical Pharmacology, Section of Neurosciences, Department of Biomedical Sciences, “San Giovanni di Dio” Hospital, University of Cagliari, Via Ospedale 54, 09124 Cagliari, Italy; Department of Medical Sciences “Mario Aresu”, University of Cagliari, Cagliari, Italy; Department of Public Health and Clinical and Molecular Medicine, University of Cagliari, Cagliari, Italy

**Keywords:** Mood disorders, Bipolar disorders, Depression, Thyroid peroxidase antibodies, Thyroid microsomal antibodies, Thyroglobulin antibodies, Hashimoto’s thyroiditis, Hashimoto’s encephalopathy, Lithium

## Abstract

Mood disorders and circulating thyroid antibodies are very prevalent in the population and their concomitant occurrence may be due to chance. However, thyroid antibodies have been repeatedly hypothesized to play a role in specific forms of mood disorders. Potentially related forms include treatment-refractory cases, severe or atypical depression, and depression at specific phases of a woman’s life (early gestation, postpartum depression, perimenopausal). With regard to bipolar disorder, studies of specific subgroups (rapid cycling, mixed, or depressive bipolar) have reported associations with thyroid antibodies. Offspring of bipolar subjects were found more vulnerable to develop thyroid antibodies independently from the vulnerability to develop psychiatric disorders. A twin study suggested thyroid antibodies among possible endophenotypes for bipolar disorder. Severe encephalopathies have been reported in association with Hashimoto’s thyroiditis. Cases with pure psychiatric presentation are being reported, the antithyroid antibodies being probably markers of some other autoimmune disorders affecting the brain. Vasculitis resulting in abnormalities in cortical perfusion is one of the possible mechanisms.

## Review

The association between thyroid function disorders and neuropsychiatric manifestations has long been known. Bauer et al. (2008) have reviewed such a relationship in patients with primary thyroid disease and primary mood disorders. The most obvious interactions are between hypothyroidism and depressive symptoms and between hyperthyroidism and manic/hypomanic symptoms. However, there may be exceptions to this simple rule.

Over the last decades, special interest has been devoted to autoimmune thyroid disease and circulating thyroid antibodies. Autoimmune thyroiditis and Graves’ disease are the two major forms of autoimmune thyroid disease. Autoimmune thyroiditis may be associated with the entire spectrum of function (overt hypothyroidism, subclinical hypothyroidism, thyrotoxicosis), but has recently been associated with neuropsychiatric manifestations even in the absence of thyroid hormone abnormalities (for a review see Leyhe and Müssig 2014).

The role of thyroid antibodies in neuropsychiatry has been investigated only recently. In fact, early studies reporting the neuropsychiatric consequences of thyroid dysfunction were not able to investigate the status of circulating thyroid antibodies, whose role might have been overlooked. One of the reasons is that, even if the commonest form of thyroiditis was first described more than one century ago by Hashimoto (1912), its autoimmune nature was discovered only in 1956 (Campbell et al. 1956) and it took several decades before methods of detection of thyroid antibodies were to be part of clinical practice, especially in psychiatry.

### Hashimoto’s thyroiditis

Hashimoto’s thyroiditis is a chronic autoimmune inflammation of the thyroid gland. The diagnosis is suspected based on the detection of elevated levels of circulating antithyroid autoantibodies. The diagnosis of thyroiditis is confirmed when fine needle aspiration biopsy, histology from thyroidectomy, or autopsy shows lymphocytic infiltration of the thyroid gland. The first to describe lymphocytic infiltration was the Japanese surgeon Hashimoto (1912), after whom the disease was named. Patients with lymphocytic thyroiditis may have various circulating autoantibodies, including antibodies against thyroid peroxidase (AbTPO), thyroglobulin (AbTG), and thyroid-stimulating hormone (TSH) receptors. Studies published until the late 1980s referred to thyroid microsomal antibodies (AbM), the fraction that turned out to be specific for AbTPO (Mariotti et al. 1987). A chronic autoimmune thyroiditis is reported by post-mortem studies in 27 % of adult women (with a peak in subjects over 50 years), and 7 % of adult men; diffuse changes are found in 5 % of women and 1 % of men (Vanderpump 2005). Hypoechoic or irregular ultrasound patterns in the presence of AbM titers ≥1:400 are considered diagnostic for Hashimoto’s thyroiditis (Marcocci et al. 1991). However, 20 % of individuals with an ultrasound pattern suggestive of thyroiditis are antibody negative (Marcocci et al. 1991). Moreover, circulating antibodies may be present in subjects with no evidence of thyroiditis (for a review see Biondi and Cooper 2008).

Even if the entire spectrum of thyroid function may be observed, Hashimoto’s thyroiditis is the most frequent cause of hypothyroidism in areas of sufficient iodine intake (Vanderpump and Tunbridge 2002; Hollowell et al. 2002). However, in its acute phase, it can cause a transient hyperthyroidism resulting from the inflammation process and the subsequent liberation of preformed thyroid hormones (Fatourechi et al. 1971). AbTG alone in the absence of AbTPO are not usually associated with thyroid dysfunction (Hollowell et al. 2002).

### Prevalence of circulating antithyroid antibodies in patients with mood disorders

Several studies have surveyed the prevalence of circulating antithyroid antibodies in psychiatric populations (principal results are summarized in Table 1). Gold et al. (1982) were the first to hypothesize that the so-called symptomless autoimmune thyroiditis may be not symptomless. Their hypothesis was based on the finding that the majority (60 %) of patients admitted to a psychiatric hospital for depression (or lack of energy) and thyroid dysfunction had circulating AbM (titer ≥1:10). It must be said, however, that patients had been diagnosed with subclinical, mild, or overt hypothyroidism, but no other evidence of thyroiditis was mentioned. Moreover, the overall prevalence of AbM in their patients was 9/100, which may be similar to the prevalence reported for the general population, especially if such low titers (≥1:10) are considered positive.

**Table 1:** Prevalence studies of thyroid antibodies in patients with mood disorders

Authors	Antibody	Bipolar disorder	Major depression	Other	Normal controls
Gold et al. (1982)	AbM	–	–	Hospitalized for depression or lack of energy 9/100 (9 %)	–
Nemeroff et al. (1985)	AbM and/or AbTG	0/3 (0 %)	7/28 (25 %)	Any hospitalized with prominent depressive symptoms 9/45 (20 %)	–
Joffe (1987)	AbM and/or AbTG	–	5/58 (8.6 %)	–	–
Haggerty et al. (1990)	AbM and/or AbTG	4/31 (13 %)	5/65 (8 %)	Non-affective disorders 7/68 (10 %)	–
Custro et al. (1994)	AbTPO and/or AbTG	–	5/9 (56 %)	Minor depression 0/66 (0 %)	1/38 (3 %)
Haggerty et al. (1997)	AbM and/or AbTG	Mixed 5/26 (19 %) Depressed 3/19 (16 %) Manic 2/51 (3.9 %)	15/218 (6.9 %)	Adjustment disorder 2/80 (2.5 %)	10/144 (6.9 %)
König et al. (1999)	AbTPO or AbM and/or AbTG	–	Severe, hospitalized 100/144 (70 %)	–	–
Kupka et al. (2002)	AbTPO	64/226 (28.3 %)	–	Any psychiatric hospitalization 324/3190 (10.2 %)	23–34/252 (9.1–13.5 %) range with different assays
Brouwer et al. (2005)	AbTPO	–	Outpatients 9/113 (8 %)	–	11/113 (10 %)
Degner et al. (2015)	AbTPO and/or AbTG	4/13 (30.8 %)	12/39 (38.5 %)	Schizophrenia 2/19 (10.5 %)	–

Despite using often the term autoimmune thyroiditis, subsequent studies focused on the mere presence of circulating antibodies. Ultrasound support was provided in some study (Custro et al. 1994), but no study provided cytological or histological evidence of thyroiditis.

Prevalence studies published in the last two decades have generally included normal controls and investigated the presence of the more specific AbTPO (Table 1). Some authors have used concentration of antibodies (or their log-transformed titers) as a continuous variable rather than the positive/negative dichotomy (Hornig et al. 1999).

The large Dutch study by Oomen et al. (1996) examined thyroid function tests, including AbTPO, in serum collected 2–3 weeks after hospitalization from 3756 psychiatric patients in 1987–1990. The prevalence of positive AbTPO was related to age and sex. The rate in the overall psychiatric sample was 331/3316 (10 %). In the subgroup older than 55 years, prevalence rates found in patients with any psychiatric hospitalization (131/968 = 13.5 %) were similar to those found in healthy individuals living in the same area and matched by age (258/1877 = 13.7 %). With regard to bipolar disorder, the Dutch study addressed some specific issues, such as lithium exposure and rapid cycling (principal data are summarized in Table 2). In particular, among 50 AbTPO positive cases, affective disorders and not other psychiatric diagnoses (dementia, schizophrenia, etc.) were overrepresented (44 %) compared with the subgroup of 83 with normal thyroid parameters (25 %). The most significant association was between antibody positivity and the subgroup with rapid cycling bipolar disorder. Rapid cycling was diagnosed in 8/45 (18 %) antibody-positive patients and in none of the 76 patients with normal thyroid parameters. The disproportion was maintained after controlling for prior treatment known to influence thyroid function, including lithium. Results contrasted with those from an earlier small study which did not reveal differences in the prevalence of circulating thyroid antibodies between 11 women with rapid cycling and 11 with non-rapid cycling bipolar disorder (Bartalena et al. 1990).

**Table 2:** Principal data from Oomen et al. (1996)

	Affective disorders	Lithium-naïve affective disorders	Rapid cycling bipolar disorder	Lithium-naïve rapid cycling bipolar disorder
Normal thyroid function	21/83 (25 %)	16/76 (21 %)	3/83 (4 %)	0/76 (0 %)
AbTPO positive	22/50 (44 %)	17/45 (38 %)	12/50 (24 %)	8/45 (18 %)

Outpatients with bipolar disorder from the Stanley Foundation Bipolar Network, a multicenter longitudinal treatment research program performed in the United States and the Netherlands (Kupka et al. 2002), were assessed for the prevalence of AbTPO and thyroid failure. The study included 226 outpatients with bipolar disorder, 252 control subjects from the general population, and 3190 psychiatric inpatients of any diagnosis. AbTPO were more prevalent (28 %) in bipolar patients than in population and psychiatric controls (3–18 %). The presence of circulating antibodies in bipolar patients was associated with thyroid failure, but not with age, gender (28.9 % of women, 27.7 % of men), current mood state (euthymia, depression, hypomania/mania, or mixed state), and rapid cycling in the past year.

In a small exploratory study of 30 major depressive patients, Fountoulakis et al. (2004) reported a significantly higher proportion of AbM in depressed patients with atypical features (according to DSM-IV) (*N* = 10) compared with healthy controls.

Leyhe et al. (2009) found that the proportion of a clinically severe degree of depressive episode was significantly higher among patients with thyroid autoantibodies (63.2 %) compared with patients with negative antibodies (28.6 %).

Degner et al. (2015) found circulating AbTPO in 17/52 (32.7 %) outpatients with uni- or bi-polar depression. The odds ratio for autoimmune thyroiditis (which was confirmed by a hypoechoic pattern in ultrasonography) was ten times higher when compared with 19 outpatients with schizophrenia.

The presence of thyroid autoantibodies was also associated with poor response to antidepressant therapy (Browne et al. 1990; Eller et al. 2010).

### Related studies

In a pilot study, Rubino et al. (2004) tested the hypothesis of a relationship between bipolar disorder and autoimmune thyroiditis by assessing three groups of women with the Serial Color-Word Test (Smith and Klein 1953). The latter consists in the analysis of reading times during the repeated confrontation with the Stroop task, i.e., the interference between reading the names and naming the colors of incongruent color-words. A discontinuous style of adaptation to the conflict situation was more marked in the group of remitted bipolar subjects compared with the group with autoimmune thyroiditis, and more marked among the latter than among non-clinical controls. Diagnosis of autoimmune thyroiditis was defined clinically with no mention of particular procedures except the presence of AbTPO.

Geracioti et al. (2003) described a patient with classic borderline personality disorder whose fluctuating mood and psychotic symptoms were directly linked to AbTG titers determined over a period of 275 days.

### Community studies

Several studies have investigated the relationships between circulating thyroid antibodies and mood disorders at the community level. In this case, data regarded principally depression. Pop et al. (1998) studied 583 perimenopausal women randomly selected from a community cohort in the Netherlands. Depression (defined as a score of 12 or higher in the self-rating Edinburgh Depression Scale) was found in 134 cases (23 %) and AbTPO in 58 cases (10 %). Multiple logistic regression analysis supported an association between positive AbTPO and depression (odds ratio 3.0; 95 % confidence interval 1.3–6.8).

Kuijpens et al. (2001) studied prospectively 310 unselected women during gestation and up to 36 weeks postpartum. The presence of AbTPO was independently associated with depression at 12-week gestation and at 4 and 12 weeks postpartum (odds ratios between 2.4 and 3.8). After the exclusion of women who were depressed at 12-week gestation or had had depression in earlier life, the presence of AbTPO during early gestation was still associated with postpartum depression (odds ratio 2.9).

The same group reported a prospective follow-up of 1017 pregnant women from the general population (Pop et al. 2006). The presence of thyroid antibodies was associated with major depression during early gestation (12 and 24 weeks) but not at the end of term, when there is maximal downregulation of the immune system.

Carta et al. (2004), in a smaller community-based study, found AbTPO in 13 of 42 (31 %) subjects with mood disorder, in 15 of 41 (37 %) with anxiety disorder, and in 19 of 139 (14 %) with no psychiatric disorder. Using multivariate logistic regression, associations were significant between thyroid antibodies and anxiety disorders (odds ratio 4.2; 95 % confidence interval 1.9–38.8) or mood disorder (odds ratio 2.9; 95 % confidence interval 1.4–6.6).

On the contrary, a large population-based study using a self-report symptom scale for depression and anxiety found no association with antithyroid antibodies (Engum et al. 2005). The prevalence of depression in individuals with positive AbTPO (115/995 = 11.6 %) did not differ from the prevalence found in the general population (385/29,180 = 13.2 %).

The role of AbTPO (independent of overt thyroid dysfunction) has also been investigated in postpartum depression in both clinical and community settings. Some studies supported an association (Pop et al. 1993; Harris et al. 1992; Lazarus et al. 1996), while others could not demonstrate it (Stewart et al. 1988; Kent et al. 1999).

### Family and twin studies

Hillegers et al. (2007) studied children of bipolar parents and found circulating AbTPO in 9 of 57 (16 %) daughters. The latter prevalence was higher than that found in matched controls (4/103 = 4 %). As the presence of antibodies was not associated with mood disorder (or any psychopathology) in the offspring, the authors suggested that offspring of bipolar subjects are more vulnerable to develop thyroid antibodies independently from the vulnerability to develop psychiatric disorders.

Vonk et al. (2007) studied 22 monozygotic and 29 dizygotic bipolar twins and 35 healthy matched control twins. Circulating AbTPO were found in 27 % of the bipolar index twins, 29 % of the monozygotic bipolar cotwins, 27 % of the monozygotic non-bipolar cotwins, 25 % of the dizygotic bipolar cotwins, 17 % of the dizygotic non-bipolar cotwins, and in 16 % of the control twins. The conclusion was that thyroid antibodies are related not only to bipolar disorder but also to the genetic vulnerability to develop the disorder. The authors proposed autoimmune thyroiditis as a possible endophenotype for bipolar disorder.

### Thyroid autoimmunity and lithium treatment

Lithium has long been known to interact with thyroid function (for reviews see Lazarus 1998; Bocchetta and Loviselli 2006). Moreover, lithium affects many aspects of cellular and humoral immunity in vitro and in vivo, but it is controversial whether lithium per se can induce thyroid autoimmunity. In a prospective study, Lazarus et al. (1986) observed significant fluctuations in antibody titer, both upwards and downwards in 10/12 patients with AbM and in 9/11 with AbTG treated with lithium for a mean of 16.2 months. The fluctuations in antibody titer are consistent with an immunomodulatory effect of lithium as has been shown in animal studies (for a review see Lazarus 1998).

Other prospective studies, although reporting fluctuations in antibody titers, failed to detect differences between pre- and post-lithium prevalence rates (Myers et al. 1985; Calabrese et al. 1985).

Prevalence of circulating thyroid antibodies among lithium-treated patients varies across studies. It is, however, important to underline once again the effects of age and sex. Initial and final prevalence rates of AbM/AbTPO and/or AbTG from our Sardinian lithium cohort followed up for 15 years (Bocchetta et al. 2001, 2007a) (women, 21–28 %; men, 4–10 %) were within the ranges observed in similar age and sex subgroups of the general population. In fact, a Sardinian survey reported an overall prevalence of AbTPO of 174/789 (22.0 %) in women and 30/444 (6.7 %) in men (Loviselli et al. 1999).

Annual incidence rates in patients after several years of lithium treatment (1.4–1.8 %) (Bocchetta et al. 2007a) did not much differ from the ranges of incidence reported for the general population, with maximum values of approximately 2 % per year in women aged over 45 (Vanderpump et al. 1995; Tunbridge et al. 1981).

As mentioned above, circulating thyroid antibodies have been found associated with affective disorders irrespective of treatment (Oomen et al. 1996).

In their prospective study, Lazarus et al. (1986) found that 16/37 (43 %) manic depressive patients had, before receiving lithium therapy, either AbM or AbTg or both.

According to Kupka et al. (2002), prevalence of circulating thyroid antibodies was not associated with prior lithium exposure. Indeed, AbTPO were found positive in 12/35 (34.3 %) patients who had never received lithium, a prevalence even higher than that found in the overall sample of bipolar outpatients (64/226 = 28 %).

In a cross-sectional study from the Berlin area, Baethge et al. (2005) did not find increased prevalence of circulating thyroid antibodies between a group of 100 adult patients with mood disorders undergoing lithium therapy (AbTPO 7/100 = 7 %; AbTG 8/100 = 8 %) and 100 age- and sex-matched controls with no history of psychiatric disorder (AbTPO 11/100 = 11 %; AbTG 15/100 = 15 %). In a prospective account of the Sardinian lithium cohort study, we reported the appearance of circulating thyroid antibodies in young subjects of both sexes within a few years of lithium exposure (Bocchetta et al. 1992). The presence of mild ultrasound thyroid abnormalities before lithium predicted the appearance of circulating antibodies (Loviselli et al. 1997). All antibody-positive lithium patients (12 women, one man) who underwent ultrasonic scan displayed a hypoechoic pattern and 11/13 (85 %) also presented a non-homogeneous echopattern; however, also the majority of antibody-negative lithium patients (31/32 = 97 % of women; 11/16 = 69 % of men) presented ultrasound abnormalities (Bocchetta et al. 1996).

Van Melick et al. (2010) found AbTPO and/or AbTG in 12/45 (27 %) lithium patients of 65 years and older, which did not differ from the prevalence found in the same age group in the general population.

Kraszewska et al. (2015) studied 66 patients (mean age, 62 years) with bipolar disorder receiving lithium for 10–44 years and found AbTPO in 30 cases (45 %) and AbTG in 43 cases (65 %).

### Hashimoto’s encephalopathy

The first description of neuropsychiatric disease associated with autoimmune thyroid dysfunction was by Brain et al. (1966). They described the case of a 40-year-old coachbuilder with known thyroid antibody-positive Hashimoto’s disease who subsequently developed focal neurological deficits and coma successfully treated with steroids and thyroxine replacement.

Subsequently, involvement of CNS in patients with thyroiditis has been reported repeatedly, resulting in the proposal of the term “Hashimoto’s encephalopathy” by Shaw et al. (1991).

Some authors have commented that there is no evidence of a pathogenic role for the antibodies, which are probably markers of some other autoimmune disorders affecting the brain (Chong et al. 2003; Fatourechi 2005). The term “steroid-responsive encephalopathy associated with autoimmune thyroiditis” (SREAT) has been proposed (Castillo et al. 2006). Clinical presentations and course vary (for a review see Marshall and Doyle 2006). Onset may be acute or subacute. Presentation may include alteration of conscious level, seizures, tremor, myoclonus, ataxia, or multiple stroke-like episodes.

Psychiatric symptoms, including depression and psychosis, have also been reported (Rolland and Chevrollier 2001; Laske et al. 2005; Mahmud et al. 2003). For a recent review on cognitive and affective dysfunctions in autoimmune thyroiditis, see Leyhe and Müssig (2014).

Course of encephalopathy may be relapsing/remitting or progressive, even evolving into dementia. Pathological EEG and non-specific imaging abnormalities may be present. Brain MRI findings may change abruptly and drastically. For example, reversible MRI lesions in the cerebral white matter, supposedly reflecting brain edema, have been reported in one case where antithyroid antibodies were also detected in the cerebrospinal fluid (Wakai et al. 2004).

To our knowledge, twelve cases have been reported to date where a prominent psychiatric presentation was associated with autoimmune thyroiditis (Table 3). The majority of cases were characterized by abnormal thyroid function (seven hypothyroidism; two hyperthyroidism), but the diagnosis of thyroiditis was supported by ultrasonography only in half the cases. In one case (Schmidt et al. 1990), thyroid hormone replacement alone resolved the mood disorder. In the two postpartum psychoses (Bokhari et al. 1998; Stowell and Barnhill 2005), antipsychotics were necessary in combination with thyroid treatment. For example, in the case with hyperthyroidism (Bokhari et al. 1998) the patient, who had presented with delusions, hallucinations, mixed mood symptoms, agitation, and transient disorientation, responded to loxapine and amoxapine, after achieving biochemical euthyroidism with propylthiouracil. In other cases, corticosteroids were also administered. For example, Mahmud et al. (2003) described the case of a 14-year-old girl who presented with a 5-year history of hallucinations and depression, elevated AbTPO, MRI white matter changes affecting the frontal lobe, and cerebral hypoperfusion shown with single-photon emission computed tomography (SPECT). The patient had significant clinical improvement and showed resolution on neuroimaging after methylprednisolone treatment. The 74-year-old woman with antidepressant-resistant depression, reported by Laske et al. (2005), who also had EEG abnormalities, was successfully treated with prednisolone as an add-on to venlafaxine therapy. The 46-year-old man reported by Liu et al. (2011), who presented with an acute depressive episode, mild diffuse cortical dysfunction on EEG, and hypothyroidism with the presence of thyroid antibodies in both serum and CSF, was successfully treated with thyroid hormone replacement and methylprednisolone.

**Table 3:** Case reports of autoimmune thyroiditis associated with mood disorder

Authors	Presentation	Sex and age	TSH (µU/ml)	Antibody titer (normal range)	Treatment
Schmidt et al. (1990)	Menstrual related mood disorder	F 37	12.1	AbM 1/1600 (<1/100) AbTG 1/10 (<1/10)	Levothyroxine
Bokhari et al. (1998)	Postpartum psychosis	F 29	<0.03	AbTPO 2330 (0–100) AbTG 597 (0–100)	Loxapine, amoxapine, propranolol, propylthiouracil
Mahmud et al. (2003)	Psychotic depression	F 14	77.4	AbTPO 6320 (0–20)	Psychotropic medication as before (valproate, sertraline, quetiapine) plus levothyroxine, methylprednisolone
Müssig et al. (2005)	Manic episode	F 32	<0.03	AbTPO 2010 (0–2) AbTG 1.1 (0–1)	Psychotropic medication as before (quetiapine, lithium, mirtazapine, venlafaxine) plus reboxetine and transcranial magnetic stimulation, carbimazole, prednisolone
Laske et al. (2005)	Severe depressive episode	F 74	Normal	AbTPO 842 (0–2)	Prednisolone, venlafaxine as before
Stowell and Barnhill (2005)	Postpartum psychotic acute mania	F 35	>150	AbTPO significantly elevated	Levothyroxine and risperidone
Tor et al. (2007)	Late-onset psychotic mania	F 72	79.4	AbTPO 50 (0–50) AbTG 2507 IU/ml (0–40)	Levothyroxine and low-dose haloperidol
Bocchetta et al. (2007b)	Affective psychosis	F 43	1.24	AbTPO>1000 (0–35) AbTG normal	Levothyroxine as before, valproate discontinuation, lithium plus olanzapine
Nagamine et al. (2008)	Lithium-induced encephalopathy	F 61	2.15	AbTPO 50 (0–0.3) AbTG 1/25,600 (1/100)	Methylprednisolone, levothyroxine as before, lithium discontinuation
Liu et al. (2011)	Acute depressive episode	M 46	66.7	AbTPO 1698 (0–5.61) AbTG 154 IU/ml (0–14.4)	Levothyroxine, methylprednisolone
Lin et al. (2011)	Manic symptoms	F 52	50.71	AbTPO 1652 (0–25) AbTG 296 IU/ml (0–25)	Levothyroxine, prednisolone, olanzapine and valproate
Lin et al. (2013)	Acute mania	F 41	18.79	AbTPO 411 (0–60) AbTG 296 IU/ml (0–60)	Levothyroxine, valproate and quetiapine

The manic episode claimed to represent the first case of bipolar disorder due to Hashimoto’s encephalopathy (Müssig et al. 2005), was associated with hyperthyroidism and pathological EEG. The patient responded to psychiatric treatment, carbimazole and short-term treatment with high doses of prednisolone.

In the subsequent cases of mania reported in association with autoimmune thyroiditis, most attention was drawn to the hypothyroid status of the patient rather than to autoimmunity.

The case of acute mania precipitated by hypothyroidism secondary to postpartum thyroiditis (Stowell and Barnhill 2005) responded to levothyroxine and risperidone. The elderly Chinese woman with late-onset psychotic mania precipitated by autoimmune hypothyroidism (Tor et al. 2007) was treated successfully with levothyroxine and low-dose haloperidol. Lin et al. (2011) reported in Taiwan a case of Hashimoto’s encephalopathy with manic symptoms that responded to levothyroxine, prednisolone in addition to olanzapine and valproate. The patient had undergone partial thyroidectomy 22 years earlier for a hyperthyroid goiter, but histology findings were not reported. A different group in Taiwan reported acute mania in a 41-year-old woman with no history of psychiatric illness. Both mania and hypothyroidism (resulting from Hashimoto’s thyroiditis as confirmed by diffusely heterogeneous and hypoechoic pattern in ultrasonography and lymphoid cell infiltration in fine needle aspiration cytology), remitted gradually within 3 weeks after treatment with levothyroxine, valproate, and quetiapine (Lin et al. 2013).

## Case series

Following our first case report of treatment-refractory affective psychosis (Bocchetta et al. 2007b), we started to collect additional cases with psychiatric presentation and autoimmune thyroiditis.

First, we provide here a follow-up of our published case (Bocchetta et al. 2007b): the patient was a 43-year-old woman, with a history of recurrent depression since the age of 31, who had developed manic, psychotic, and soft neurological symptoms across the previous 3 years in concomitance with her first diagnosis of Hashimoto’s thyroiditis. Brain MRI had evidenced several non-active lesions in the white matter from both hemispheres, suggestive of a non-specific past vasculitis. Brain SPECT had showed cortical perfusion asymmetry particularly between frontal lobes. Nailfold videocapillaroscopy had evidenced a reduced number of capillaries and an irregularity in pattern, consistent with past vasculitis. Over the following 10 years, the patient has been maintained with a combination of lithium, olanzapine, bupropione, citalopram, and benzodiazepines. Functioning has improved although not returning to pre-morbid levels. In particular, she has not manifested psychotic symptoms any more and her suicidality has gradually disappeared. Current status is characterized by mild anxiety and impairment in memory retrieval. The latter cognitive impairment remained unchanged during and after 4-week therapy with methylprednisolone (maximum dose 16 mg/daily) prescribed by the dermatologist for recurrence of her urticaria vasculitis. Thyroid function has been maintained adequately with levothyroxine replacement. AbTPO titers have remained elevated (last measurement >1000 IU/ml; normal range 0–35 IU/ml) with normal AbTG.

The series summarized in Table 4 regards patients visited at our psychopharmacology unit over the last 20 years and is limited to cases with antibody titers clearly exceeding the upper end of the normal range. The diagnosis of thyroiditis was supported by ultrasonic scan. In two cases, the diagnosis of thyroiditis was confirmed with cytology by fine needle aspiration biopsy (case # 8) or histology after thyroidectomy (case # 1).

**Table 4:** Case series

#	Psychiatric diagnosis, (age at onset)	Sex (current age)	Age at first measurement of circulating antibodies	Antibody titer when first detected (normal range)	Thyroid diagnostic and therapeutic procedures	Treatment	Notes
1	Bipolar I (41)	F (70)	50	AbM 1:1600 AbTPO 397 (0–100)	Thyroidectomy at age 63 (histology: multinodular goiter plus chronic lymphocytic thyroiditis) Levothyroxine replacement since thyroidectomy	Poor response to 1 year of lithium. Partial response to valproate (age 52–70)	Cognitive impairment, limb and head tremor, MRI abnormalities Excision of nodule at age 20 (no histology available)
2	Bipolar II (23)	F (76)	54	AbTPO 1661 (0–20) AbM 1:102,400 AbTG 1:100	Non-homogeneous echopattern. Levothyroxine for hypothyroidism since age 54	Lithium response for 21 years (age 42–63), then poor response	Hand and leg tremor, cognitive decline to dementia, MRI and SPECT abnormalities
3	Schizoaffective (23)	F (41)	25	AbTPO 1567 (0–20)	Non-homogeneous hypoechoic echopattern Levothyroxine for hypothyroidism since age 25	Poor response to 1 year of lithium. Partial response to clozapine (age 25–41)	Recurrent auditory hallucinations, SPECT abnormalities
4	Bipolar II (45)	F (74)	51	AbM 1188 (0–50) AbTG 829 (0–50)	Echopattern of thyroiditis plus nodules at age 51 TSH-suppressive levothyroxine for goiter since age 51	Excellent response to lithium (age 46–74)	Head tremor Suspected thyroid dysfunction during adolescence
5	Recurrent major depression (38)	F (87)	61	AbM 1:65,536 AbTPO 6393 (0–200)00	Diffuse non-homogeneous echopattern plus multiple nodules No thyroid treatment	Excellent response to lithium (age 57–87)	Head tremor.
6	Bipolar I (39)	F (64)	61	AbTPO 255 (0–35)	Hyperthyroidism after lithium withdrawal, treated with methimazole Diffuse non-homogeneous echopattern plus multiple nodules	Good lithium response (age 49–59). Lithium + haloperidol between age 61 and 64	AbTPO >2000 (0–35) at age 62, 1 year after new lithium prescription
7	Chronic depressive schizoaffective disorder (17)	F (46)	30	AbTPO positive (titer not available)	Echopattern of thyroiditis (not otherwise specified) Levothyroxine for hypothyroidism since age 35	Poor response to first-generation antipsychotics and carbamazepine. Partial response to clozapine (age 30–40). Partial response to clozapine + lithium (age 40–46)	AbTPO 3150 (0–35) at age 40 SPECT abnormalities
8	Depressive schizoaffective disorder (26)	F (48)	40	AbM 1:25,600	Non-homogeneous echopattern plus multiple nodules. Fine needle aspiration consistent with Hashimoto’s thyroiditis Levothyroxine for hypothyroidism since age 42	Partial response to lithium (age 32–48)	AbTPO 7757 (0–200) at age 42 and 655 (0–20) at age 45 Tremors of hands, legs, mouth and tongue
9	Psychotic bipolar I (23)	F (54)	37	AbM 1:1600 AbTG negative	Echopattern consistent with thyroiditis (not otherwise specified) Levothyroxine for hypothyroidism since age 39	Good response to lithium (age 37–54)	Highest AbTPO titer 862 (0–20) Autoimmune polyendocrine syndrome first diagnosed at age 50
10	Psychotic bipolar I (24)	M (72)	48	AbTPO 578 (0–200) AbM 1:6400 AbTG 1:400	Subclinical hypothyroidism and hyperthyroidism between age 43 and 48. Diffuse non-homogeneous echopattern	Excellent response to lithium (age 35–53) and subsequently to carbamazepine (age 53–72) after diagnosis of renal failure	Highest AbTPO titer 315 (0–20) EEG abnormalities at age 43
11	Schizoaffective disorder (24)	F (43)	31	AbTPO 172 (0–10) AbM 1:6400 AbTG negative	Echopattern consistent with thyroiditis (not otherwise specified) Levothyroxine for hypothyroidism since age 33	Poor response to lithium + carbamazepine (poor adherence to treatment)	Normal antibodies at age 28 Highest AbTPO titer 3063 (0–120)
12	Bipolar schizoaffective disorder (33)	F (57)	49	AbTPO 4260 (0–100) AbTG 100 IU/ml (0–100)	Echopattern consistent with thyroiditis (not otherwise specified) Levothyroxine for hypothyroidism since age 49	Partial response to lithium + haloperidol (age 38–57)	AbTPO titer 241 (0–10) with AbM 1:25,600 at age 53 Suspected Sjögren syndrome at age 56
13	Bipolar I (38)	F (49)	46	AbTPO 1072 (0–9) AbTG 65 (0–4)	Echopattern consistent with thyroiditis (not otherwise specified) Levothyroxine for hypothyroidism since age 46	Partial response to lithium + quetiapine (age 46–49)	Multiple sclerosis first diagnosed at age 20.
14	Psychotic bipolar I (23)	F (60)	44	AbTPO 514 (0–35)	Non-homogeneous echopattern plus multiple nodules Levothyroxine for hypothyroidism since age 44	Partial response to valproate (age 51–58). Good response to valproate + lithium (age 58–60)	Celiac disease first diagnosed at age 44

In some cases, further diagnostic procedures were undertaken, especially when (a) response to psychiatric treatment was poor; (b) cognitive or neurological symptoms were prominent; and (c) in attempts at investigating potential pathogenic links. The following details regard cases with noteworthy findings.

### Case # 1

A 65-year-old woman was referred to our unit for monitoring of valproate therapy. Lithium treatment had been stopped after 1-year trial because the patient had used a lithium overdose in one of her many suicide attempts. She had undergone thyroidectomy 2 years earlier and was under adequate replacement with levothyroxine. Response to valproate had been partial and she was on a combined therapy with antipsychotics. Given the presence of cognitive impairment and limb and head tremor, we requested brain MRI. White matter hyperintensities consistent with microangiopathy were evidenced close to the frontal horns and in the body of lateral ventricles, in subcortical right parietal and left frontal and occipital regions, in the left thalamus and the semioval center. Initial enlargement of CSF spaces was also reported. Antinuclear antibodies were also evidenced (titer 1:160; normal range <1:80). Over the following 5 years, she manifested recurrent episodes of depressed mood, time and place disorientation, wandering, impairment in memory. She also manifested postural and gait instability, diffused tremor of head and limbs.

### Case # 2

**Fig. 1:**
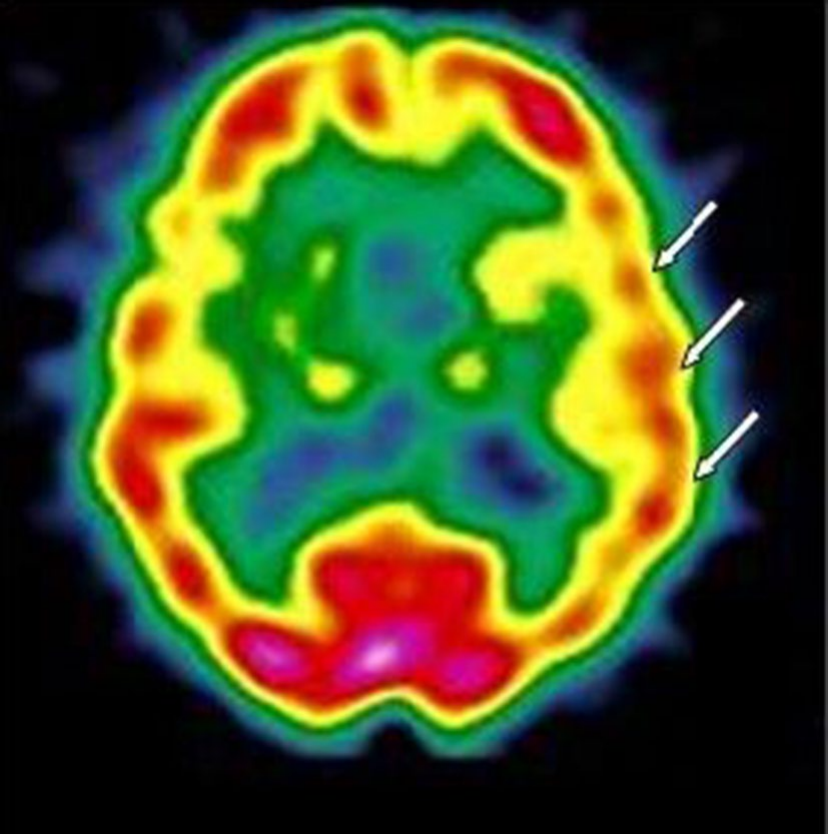
Case # 2. ^99m^Tc-ECD brain SPECT: hypoperfusion in the left temporal and parietal region (*arrows*)

A 54-year-old woman was referred to our unit for monitoring of lithium therapy. She had been treated successfully with lithium since the age of 42, but thyroid function had never been examined. We requested a thyroid workup resulting in the diagnosis of autoimmune hypothyroidism. Over the next 9 years, the patient complained of impairment in concentration and memory and hand and leg tremor. AbTPO titer was invariably elevated (around 2000 IU/ml; normal range 0–20 IU/ml). At age 63, the patient abandoned lithium maintenance, considering it responsible for the worsening of tremors. Over the subsequent 2 years, mood fluctuations become more severe and the patient was treated with antidepressants and olanzapine. She began to manifest dysarthria and her resting tremor worsened. Cognition was further deteriorated. She was hospitalized and brain MRI evidenced widespread encephalopathy regarding periventricular white matter. The patient was again referred to our lithium clinic with a view to lithium’s potential neuroprotective properties. Over the following 2 years, there was a partial stabilization of mood fluctuations with low-dose lithium, but cognitive function worsened. Brain MRI confirmed prior evidence of white matter encephalopathy, with atrophic widening of ventriculi, CSF spaces, cisternal and cortical sulci, especially in the frontal cortex. At age 69, the patient manifested anomia and impairment in concentration and attention. She had a score of 12/30 on the mini-mental state examination (MMSE). Instrumental activities of daily living (IADL) and activities of daily living (ADL) were markedly impaired, with scores of 2/8 and 4/6, respectively. EEG evidenced marked and widespread slow bioelectric abnormalities. Brain SPECT with ^99m^Tc-ethyl cysteinate dimer (ECD) evidenced non-homogeneous cortical perfusion of both hemispheres, regarding particularly left temporal cortex and parietal cortex (Fig. 1). Nailfold videocapillaroscopy evidenced a “flou” effect (widespread reduced visibility of capillaries due to microedema). Capillary loops were hypodense or atypical, with meandering and dilatation especially at the top. Elongated capillaries were also frequent and sludge was also present. The report concluded that the microcirculatory abnormalities were consistent with a non-specific organic microangiopathy. We suspected an immune-mediated vasculitis and prescribed a trial with corticosteroids. However, treatment was abandoned after a few weeks given the onset of diabetes. Cognitive symptoms worsened over the following 7 years resulting in severe dementia. Lithium has been maintained in combination with quetiapine with a partial effect on mood swings and behavioral manifestations.

### Case # 3

**Fig. 2:**
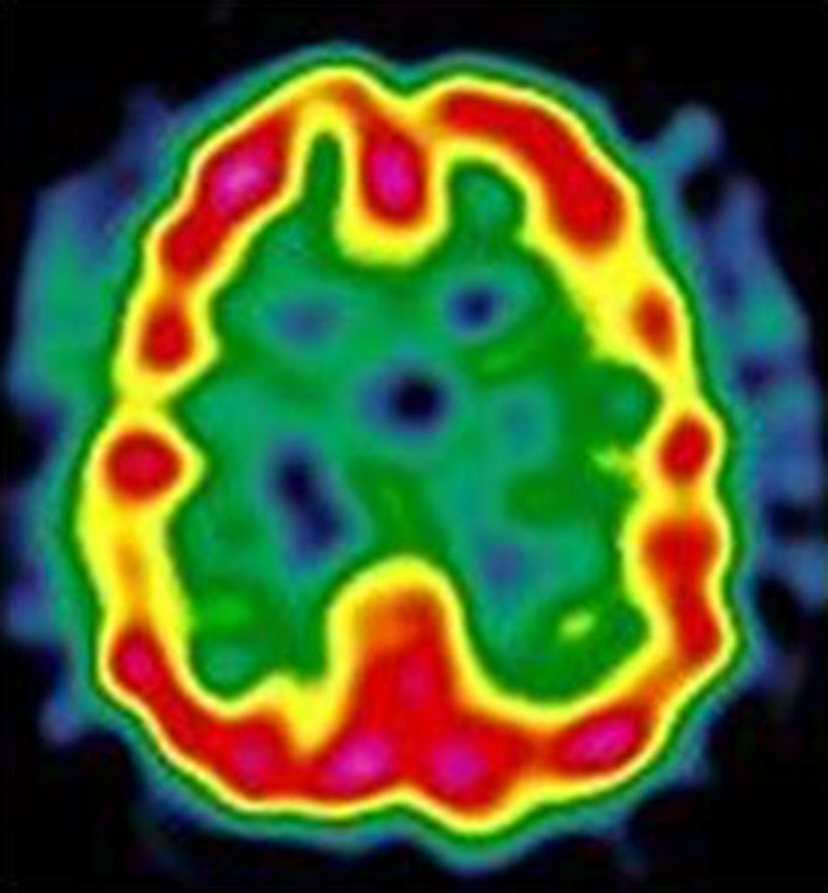
Case # 3. ^99m^Tc-HMPAO brain SPECT: non-homogeneous tracer uptake in the cortex of both hemispheres

The patient had suffered from a first psychotic episode characterized by auditory hallucinations and persecutory delusions at the age of 23. Her symptoms subsided with haloperidol, but she manifested extrapyramidal side effects. Following a new psychotic manic episode, the patient was referred to our lithium clinic at the age of 25. Thyroid workup revealed mildly increased TSH (5.30 µIU/ml), a diffused goiter, and Hashimoto’s thyroiditis (AbTPO titer = 1567 IU/ml; normal range 0–20 IU/ml). She was prescribed L-thyroxine substitution. After an apparent initial response to lithium, the patient developed a new psychotic manic episode. As auditory hallucinations did not respond to first-generation antipsychotics, she was prescribed treatment with clozapine. Over the following 18 years, the patient has been maintained with clozapine (100–150 mg/die), augmented with small doses of haloperidol to counteract the occasional reemergence of auditory hallucinations. She has continued l-thyroxine substitution and thyroid hormones have been within the normal range, but AbTPO remained elevated (>1000 IU/ml; normal range 0–20 IU/ml). Ultrasonic scans of the thyroid gland evidenced a progressive reduction of goiter, whereas the echopattern was consistent with chronic autoimmune thyroiditis (non-homogeneous, hypoechogenic, showing a fibro-connective plot with pseudonodular features). We suspected the presence of vasculitis and requested further investigation. Nailfold videocapillaroscopy evidenced a reduction in the number of capillaries, with a non-homogeneous pattern. In particular, the tip was the only part visible in one-third of capillaries. Brain SPECT with ^99m^Tc-HMPAO (hexamethylpropyleneamine oxime) evidenced non-homogeneous cortical perfusion of both hemispheres, with no specific area of clear hypoperfusion (Fig. 2).

### Case # 4

The patient had suffered from a first depressive episode at the age of 45. One year later, following an antidepressant-related switch into hypomania, she was prescribed lithium. She was referred to our lithium clinic at the age of 51. Medical history revealed that the patient had also suffered from head tremor for many years and had experienced marked weight loss during adolescence. Her basal metabolic rate had been studied (it was the only method used to investigate thyroid function at that time), and found normal. We requested an updated thyroid workup that resulted in a diagnosis of euthyroid autoimmune thyroiditis. In any case, the endocrinologist prescribed TSH-suppressive levothyroxine treatment. Response to lithium has been excellent for the following 23 years. AbTPO titer has risen up to >1000 IU/ml (normal range 0–35 IU/ml). Head tremor is still evident.

### Case # 5

The patient had started lithium therapy at the age of 57 and response was excellent. She was referred to our lithium clinic at the age of 59. Thyroid function had never been examined. We requested a thyroid workup resulting in the diagnosis of autoimmune thyroiditis. Despite her very high antibody titers, thyroid function was normal, and no treatment was indicated. Lithium concentrations were maintained below 0.5 mmol/l over the following 28 years, given the presence of head tremor and bradycardia. The patient has remained euthyroid without any specific treatment. Her head tremor is still present.

### Case # 6

The patient had been maintained well with lithium from the age of 49 up to the age of 59. Then, the patient abandoned lithium prophylaxis worried about the presence of a multinodular goiter. Thyroid hormone concentrations had been normal during lithium therapy, but hyperthyroidism emerged after lithium withdrawal. She was treated with methimazole. Thyroid hormone concentrations normalized in a few months and antithyroid treatment was stopped. One year later, at age 61, mood fluctuations became prominent and the patient was referred to our lithium clinic. Given the presence of suicidality, we prescribed lithium again. During the following 3 years, mood fluctuations were attenuated with a combined treatment (lithium, low-dose haloperidol, and benzodiazepines). The patient has no longer experienced suicidal thoughts. AbTPO titer has repeatedly been found >2000 IU/ml (normal range 0–35 IU/ml), with normal thyroid hormone concentrations without any other endocrinological treatment.

### Case # 7

**Fig. 3:**
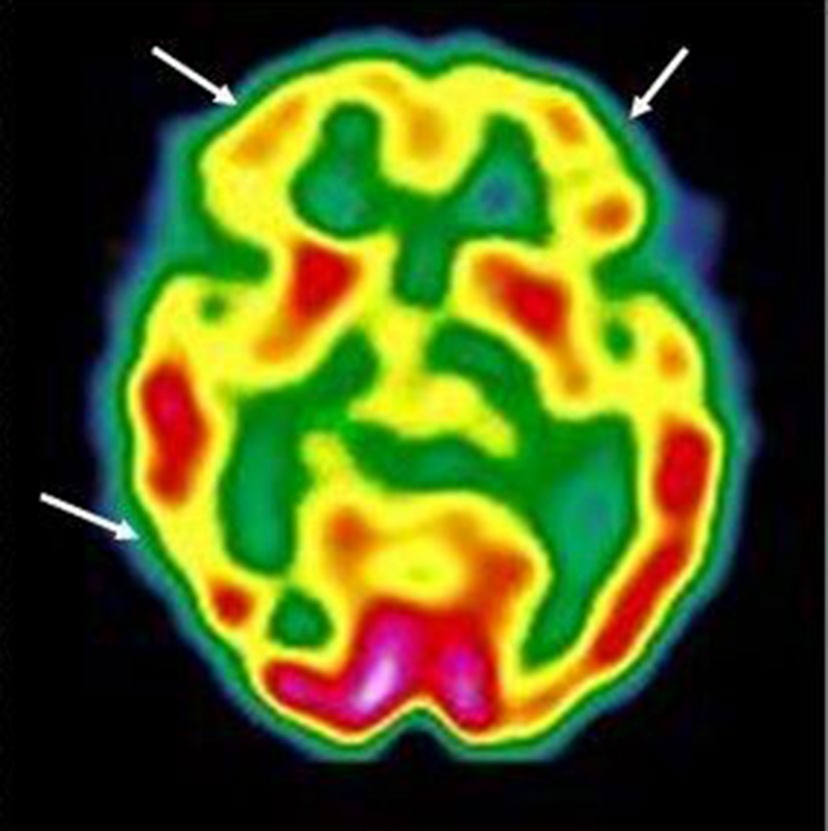
Case # 7. ^99m^Tc-ECD brain SPECT: decreased tracer uptake in both frontal lobes and in the right occipital region (*arrows*)

The patient had suffered from chronic depressive schizoaffective disorder since the age of 17. She had failed to respond to adequate trials with various antipsychotics, alone or in combination with carbamazepine. Then, at the age of 30, she was referred to our unit. Over the last 2 years, treatment had consisted in risperidone and carbamazepine. Her presentation included auditory hallucinations, persecutory and religious delusions, bizarre and aggressive behavior. Negative symptoms were also present. The medical workup resulted in the diagnosis of euthyroid Hashimoto’s thyroiditis. Psychotropic treatment was gradually changed to clozapine (up to 375 mg/daily). Negative symptoms and behavioral manifestations improved, but auditory hallucinations persisted. Clozapine doses were progressively reduced due to the emergence of obsessive–compulsive symptoms. At age 35, because of increasing TSH concentrations, she was prescribed levothyroxine. At age 40, the patient manifested exacerbation of psychosis with concomitant depressive symptoms and suicidal thoughts. We suspected the presence of vasculitis associated with her thyroiditis (AbTPO titer = 3150 IU/ml; normal range 0–35 IU/ml), and requested further investigation. Nailfold videocapillaroscopy evidenced meandering and dilatation of capillaries, slowed flux and “flou” effect (reduction in the visibility of the capillary bed due to microedema). Brain SPECT with ^99m^Tc-ECD evidenced non-homogeneous cortical perfusion regarding particularly the right occipital lobe and both frontal lobes (Fig. 3). We ruled out treatment with corticosteroids for several reasons, including the evidence of reduced bone mineral intensity on densitometry. Given the history of suicide attempts, we opted for adding lithium to clozapine. The patient has been maintained for the last 6 years with a combination of lithium and clozapine (current dose 100 mg/daily). Behavioral and affective symptoms have improved and the patient has no longer experienced suicidal thoughts. Auditory hallucinations have persisted, but the patient is less preoccupied perhaps because she attributes them to her impaired perfusion of the brain.

### Case # 9

After the diagnosis of autoimmune thyroiditis at age 37, AbTPO titers remained elevated over the following 17 years, reaching the highest titer of 862 IU/ml (normal range 0–20 IU/ml). At age 50, the patient was diagnosed with an autoimmune polyendocrine syndrome characterized by thyroiditis, diabetes, and coeliac disease with the presence of anti-reticulin, anti-gliadin, and anti-endomysium antibodies.

### Case # 14

According to available clinical records, Hashimoto’s thyroiditis had been first diagnosed at the age of 44. At the same time, coeliac disease was also diagnosed based on the presence of anti-gliadin antibodies. Replacement with levothyroxine was started at age 45 but did not appear to improve mood disorder. After several years of treatment with antidepressants and antipsychotics, valproate was prescribed at the age of 51. However, given the presence of recurrent suicide ideation, lithium was eventually combined with valproate at the age of 58 with good response over the last 3 years.

## Discussion

The hypothesis of an interaction between thyroid antibodies and mood disorders was first raised in the early 1980s. Early studies investigated the prevalence of circulating antibodies in sample of patients with depressive disorder. Sample size was generally limited and cut-off for antibody positivity was rather low (1:10). Such low titers have been associated with the development of thyroiditis in prospective community studies (Tunbridge et al. 1981), but they are very prevalent in the general population and their concomitant occurrence with other common conditions (including mood disorders) may be obviously due to chance.

Moreover, such early prevalence studies did not include controls, which is a relevant pitfall. In fact, prevalence rates vary widely in the general population, depending on several reasons, including sex and age distribution, geographic origin, not to mention variations in the cut-off for antibody positivity (Vanderpump et al. 1995; Hollowell et al. 2002; McGrogan et al. 2008).

The overall prevalence in an Italian survey (using a cut-off of 1:100 for AbM and/or AbTG) was 12.6 % (females, 17.3 %; males, 7.0 %), and there was a progressive increase with age (from 2.4 % in 1–14-year-old children to 21.9 % in the 46–55-year range, with little change in older subjects) (Aghini-Lombardi et al. 1999). The reported prevalence in the United States healthy population (using a competitive immunoassay procedure) was 13 % for AbTPO (≥0.5 IU/ml) and 11.5 % for AbTG (≥1.0 IU/ml) (Hollowell et al. 2002).

Hornig et al. (1999) who studied a sample of patients with affective disorders for various autoantibodies (antinuclear, anti-double stranded DNA, anticardiolipin, and antithyroid) warned of the potentially misleading influence of age and sex in prevalence studies.

With regard to antithyroid antibodies and thyroiditis, middle-aged women are known to show the highest prevalence rates (Vanderpump 2005). Sex differences in thyroid dysfunction are very relevant even with regard to the diagnosis and treatment of mood disorders, as recently reviewed by Bauer et al. (2014).

Overall, the association of thyroid circulating autoantibodies with not otherwise specified mood disorders cannot be considered clearly established. The discrepancies across studies may depend on the heterogeneity of samples with regard to age, sex, ethnicity, study setting, or severity of the mood disorder. On the contrary, clinical samples and community studies have begun to reveal increased prevalence of circulating thyroid antibodies in the following forms of mood disorders: treatment-refractory cases (Browne et al. 1990; Eller et al. 2010), severe (Custro et al. 1994; König et al. 1999; Leyhe et al. 2009) or atypical depression (Fountoulakis et al. 2004), and depression at specific phases of a woman’s life (early gestation, postpartum depression, perimenopausal) (Pop et al. 1993, 1998, 2006; Kuijpens et al. 2001; Harris et al. 1992; Lazarus et al. 1996).

With regard to bipolar disorder, sample size was small in several studies from clinical settings (Nemeroff et al. 1985; Haggerty et al. 1990; Bartalena et al. 1990; Degner et al. 2015), whereas community studies are not the proper instrument for bipolar disorder. In fact, as underscored by Carta and Angst (2005), manic/hypomanic episodes may be missed in epidemiological surveys, especially when the subject is the only source of information.

The first suggestion of a potential association between bipolar spectrum disorders and circulating thyroid antibodies was published by Haggerty et al. (1997), who reported increased prevalence of AbM and/or AbTG in specific subgroups (rapid cycling, mixed, or depressive bipolar) compared with healthy individuals controlled for age and sex.

One study of a large bipolar sample supported the association of circulating AbTPO with rapid cycling (Oomen et al. 1996). Kupka et al. (2002) found an excessive prevalence of circulating AbTPO (28 %) in their overall bipolar sample (*N* = 226) compared with controls. The high prevalence of thyroid antibodies found in bipolar men (27.7 % compared with 28.9 % in women) represents a rare exception to the female predominance.

The following two other lines of evidence support the relationship between thyroid antibodies and bipolar disorder: (a) in the offspring of bipolar subjects, the vulnerability to develop thyroid autoimmunity appears to be independent from the vulnerability to develop psychiatric disorders (Hillegers et al. 2007); (b) the twin study (Vonk et al. 2007) suggests autoimmune thyroiditis as a possible endophenotype for bipolar disorder. It must be underscored that the diagnosis of thyroiditis in the latter two studies was not supported by further diagnostic procedures apart from the presence of circulating antibodies.

### “Hashimoto’s mood disorders”?

Despite the uncertainty of their role, the presence of thyroid antibodies in severe cases of affective disorders cannot be disregarded. Müssig et al. (2005) claimed that their report represents the first case of bipolar disorder due to Hashimoto’s encephalopathy. In our own published case report, Hashimoto’s thyroiditis was suspected to be involved in the deteriorating course of bipolar affective psychosis (Bocchetta et al. 2007b). At that time, we speculated that the rare severe cases of Hashimoto’s encephalopathy presenting with mood disorder may represent only the tip of an iceberg. Our series of heterogeneous cases reported here is just an indication that autoimmune thyroiditis may be frequently observed in concurrence with bipolar spectrum disorders. This does not necessary mean that thyroid antibodies play a direct role.

We speculate that there may be a wide spectrum of possibilities, varying from typical cases of mood disorders with full response to treatment to treatment-resistant cases and even to cases evolving into dementia. Obviously, the role of antibodies is more suspected in severe cases. Given the nature of our unit, the majority of cases regarded patients treated with lithium. In our lithium clinic, we have included the search for thyroid antibodies in the panel of laboratory tests since the late 1980s. At that time, specific methods became routinely available and we had realized the relevance of thyroid autoimmunity in lithium-related hypothyroidism (Bocchetta et al. 1991).

In older patients from this series, the time relationship between onset of thyroiditis, onset of affective disorder, and treatment with lithium cannot be assessed. On the other hand, more recent cases (#7, #9, and #14) witness that thyroiditis may occur before lithium exposure.

The possible transition from “Hashimoto’s mood disorders” to “Hashimoto’s encephalopathy” is just a matter of speculation. If one assumes that there is a direct role of thyroid antibodies in CNS, any presentation is possible from a mere psychiatric one to severe life-threatening neurologic encephalopathy.

However, the acceptance of a new disease entity is usually cautious. The communities of neurologists and endocrinologists are still doubtful regarding the entity Hashimoto’s encephalopathy (Chong et al. 2003; Fatourechi 2005). We speculate that the community of psychiatrists will be even more cautious. In his paper entitled “Hashimoto’s encephalopathy: myth or reality? An endocrinologist’s perspective,” Fatourechi (2005) stated: “Since the first description of a case of episodic encephalopathy associated with Hashimoto’s thyroiditis in 1966, many cases of corticosteroid-responsive encephalopathy associated with positive antithyroid antibodies, clinical Hashimoto’s thyroiditis, or spontaneous autoimmune thyroid failure have been reported. These patients have neurologic manifestations of encephalopathy unrelated to other known causes. The condition has thus been termed ‘Hashimoto’s encephalopathy’. The literature shows no proven association between thyroid disease and the neurologic process. Although the association of a common endocrinologic condition and a rare neurologic disease may occur by chance, this type of encephalopathy probably has an autoimmune nature and thus is more likely to occur in the background of another autoimmune condition such as autoimmune thyroid disease. Until the pathogenesis of these coincident conditions is better defined, the term ‘corticosteroid-responsive encephalopathy associated with autoimmune thyroiditis’ is more accurate and descriptive than Hashimoto’s encephalopathy. Advances in the field may clarify this seemingly inconsistent terminology.”

A decade later, the same conclusion can be applied to cases with prominent psychiatric presentation, as those described in this paper. In any case, even the term SREAT must be considered provisional as it represents an example of “criterium ex adiuvantibus” which refers to the process of making an inference about disease causation from an observed response of the disease to a treatment. It must be noted that, both in the psychiatric (this review) and in the neurological setting [see, for example, Katoh et al. (2007) for a case with antibodies against the aminoterminal region of alpha-enolase], there have been cases with autoimmune thyroiditis which resolved without undergoing corticosteroid therapy and there may have been a publication bias against steroid-non-responsive cases.

### Diagnostic procedures

The procedures in both previous case reports and in this case series have not been systematic, as they have reflected the availability of methods over the last two decades. Procedures might have also been influenced by the appearance of relevant literature reports. An example is the replacement of ^99m^Tc-ECD with ^99m^Tc-HMPAO as SPECT tracers.

It must be said that, by contrast with the severe cases of Hashimoto’s encephalopathy, pure psychiatric presentation may result in lack of further diagnostic procedures. An exception may be represented by treatment-refractory mood disorders or apparently unusual associations (mania with hypothyroidism).

In our own published case report (Bocchetta et al. 2007b) brain SPECT showed cortical perfusion asymmetry particularly between frontal lobes.

Nagamine et al. (2008) reported a case of Hashimoto’s encephalopathy induced by lithium in a woman with bipolar II disorder and history of lithium-induced thyrotoxicosis associated with silent thyroiditis. SPECT detected multiple areas of hypoperfusion, particularly in frontal, parietal, and occipital cortices. Encephalopathy resolved with methylprednisolone therapy.

However, it is noteworthy that cortical hypoperfusion is also found in brain SPECT imaging from series of patients with euthyroid autoimmune thyroiditis, even in the absence of any treatment or clinical evidence of CNS involvement (Zettinig et al. 2003; Piga et al. 2004). Various degrees of defective tracer uptake have been observed in the three patients from this series who underwent brain SPECT (# 2, # 3, # 7), with an apparent correlation with the severity of the psychiatric disorder.

Another study reported parietal SPECT asymmetry in depressed patients in the presence of thyroid autoimmunity (Hardoy et al. 2011).

Pilhatsch et al. (2014) studied positron emission tomography with ^18^F-fluorodeoxyglucose in hypothyroid patients with autoimmune thyroiditis suffering from neuropsychiatric symptoms: serum levels of AbTG were significantly correlated with glucose metabolism in the perigenual anterior cingulate cortex, a brain region previously shown to regulate affect and emotional homeostasis.

### Potential mechanisms

The mechanisms involved in mood disorders associated with autoimmune thyroiditis are only a matter of speculation. Even with regard to cases of encephalopathy with severe neurological presentation, there is no evidence of a pathogenic role for the antibodies, which are probably markers of some other autoimmune disorders affecting the brain. Nevertheless, antithyroid antibodies were in some cases detected in the cerebrospinal fluid (Ferracci et al. 2003; Wakai et al. 2004). The focal and global cerebral involvement has also been attributed to autoimmune-mediated cerebral vasculitis, with or without immune complex deposition (Kothaner-Margeiter et al. 1996; Shaw et al. 1991), or to an anti-neuronal antibody-mediated mechanism (Takahashi et al. 1994). Others view it as a non-vasculitic autoimmune inflammatory meningoencephalitis (Castillo et al. 2006). Blanchin et al. (2007) detected high levels of AbTPO in the cerebrospinal fluid of patients with Hashimoto’s encephalopathy. Both sera from their patients and monoclonal AbTPO were able to bind monkey cerebellar cells. Moreover, monoclonal AbTPO reacted with normal human astrocytes from primary cultures. Moodley et al. (2011) described the presence of antigenic targets for anti-TSH-receptor IgG on human cortical neurons and anti-TG IgG in cerebral vasculature.

We have hypothesized that abnormalities in cortical perfusion, as seen with SPECT, may represent a pathogenic link between thyroid autoimmunity and mood disorders (Bocchetta et al. 2007b). Peripheral microvasculitis may be evidenced with nailfold capillaroscopy, which is consistent with the view that systemic inflammation might contribute to endothelial dysfunction in patients with autoimmune thyroiditis (Taddei et al. 2006).

Yoneda et al. (2007) have suggested that antibodies against the amino terminal region of alpha-enolase play a role in Hashimoto’s encephalopathy. Because alpha-enolase is expressed in vascular endothelial cells, autoantibodies against this enzyme may be associated with vasculitis.

Drexhage et al. (2011) reported a lack of anti-inflammatory T cell forces in patients with bipolar disorder who were also positive for thyroid autoimmune disease.

We would like to mention another similar autoimmune disorder involving CNS that has recently drawn the attention and whose presentation may be neurological and/or psychiatric. This is associated with antibodies against subunits of the *N*-methyl-d-aspartate receptor (AbNMDAR) (for a review see León-Caballero et al. 2015). AbNMDAR have been involved in encephalitis (Kayser et al. 2013) and in mania (Dickerson et al. 2012), and have been found in excess among patients with different psychiatric disorders (schizophrenia, schizoaffective, disorder, bipolar disorder, major depressive disorder) (for a meta-analysis see Pearlman and Najjar 2014).

Interestingly, anti-NMDAR has also been found in psychiatric patients with circulating antithyroid antibodies (Chiba et al. 2013).

Finally, it is noteworthy that a genetic association has been found between bipolar disorder and polymorphism of Toll-like receptor 4 gene, which plays a major role in innate immunity (Oliveira et al. 2014). Moreover, in a stratified analysis, two alleles were found more prevalent in bipolar patients with autoimmune thyroiditis. The authors suggested that an increased susceptibility to infections and/or autoimmunity may be a mechanism underlying a subgroup of bipolar disorders.

### Treatment

The first objective in the treatment of patients with autoimmune thyroiditis is to ensure a euthyroid status. The presence of circulating thyroid antibodies in itself does not require any treatment. In our experience, long-term treatment with levothyroxine may result in negativization of antibodies. However, the latter event is exceptional, especially when titers are as high as in our case series. Therefore, we cannot provide any support to the hypothesis that mood symptoms improve while antibody levels normalize.

In autoimmune thyroiditis presenting with mood disorders (Table 3), thyroid hormone substitution has been used in case of hypothyroidism (Schmidt et al. 1990; Mahmud et al. 2003; Stowell and Barnhill 2005; Tor et al. 2007; Liu et al. 2011; Lin et al. 2013) or antithyroid drugs in case of hyperthyroidism (Bokhari et al. 1998; Müssig et al. 2005).

It is noteworthy that treatment-resistant depression may respond to adjunctive therapy with thyroid hormones (for a review see Bauer et al. 2008). In patients with bipolar disorder, administration of supraphysiologic thyroid hormone has been suggested to improve depressive symptoms by modulating function in components of the anterior limbic network (Bauer et al. 2015).

Cases of mania associated with hypothyroidism due to thyroid autoimmunity have been reported to respond to hormone substitution in combination with common antimanic drugs (Stowell and Barnhill 2005; Tor et al. 2007; Lin et al. 2013).

Cases of Hashimoto’s encephalopathy with severe neurological symptoms may require immunotherapy (corticosteroids, immunoglobulins, plasmapheresis) (for a review see Schiess and Pardo 2008). In cases with psychiatric presentation (Table 3), corticosteroids may be necessary as well (Mahmud et al. 2003; Mussig et al. 2005; Laske et al. 2005; Nagamine et al. 2008; Liu et al. 2011; Lin et al. 2011).

Cummings et al. (2007) reported a case of Hashimoto’s encephalopathy with neurological and psychotic symptoms in a 19-year-old man who underwent neuropsychological testing before, during, and after steroid treatment. Behavioral and psychotic symptoms remitted before cognitive deficits, suggesting that the latter may be more appropriate for guiding the duration of steroid treatment.

Nevertheless, potential corticosteroid-induced depression, hypomania, or overt psychosis is to be taken into account (for a review on corticosteroid-induced neuropsychiatric disorders, see Bhangle et al. 2013).

We have no data about response to steroids, except for the brief concurrent trial with methylprednisolone prescribed by the dermatologist for recurrence of urticaria vasculitis during follow-up of our published case and a trial with corticosteroids (in case # 2 from this series) that was abandoned after a few weeks because of the onset of diabetes.

With regard to lithium treatment, circulating thyroid antibodies should be checked besides the common panel of thyroid function tests, as they are one of the risk factors for lithium-induced hypothyroidism (Bocchetta et al. 2001; Kupka et al. 2002). As already mentioned, lithium effects on thyroid function have long been known (for reviews see Lazarus 1998; Bocchetta and Loviselli 2006).

It must also be said that lithium has been considered responsible for a case of Hashimoto’s encephalopathy (Nagamine et al. 2008). Nevertheless, we reported that lithium can improve the course of a bipolar case with refractoriness possibly associated with autoimmune thyroiditis (Bocchetta et al. 2007b). In any case, lithium is recommended in case of high suicide risk (for a review see Lewitzka et al. 2015). Interestingly, lithium possesses, among other properties, a regulatory effect of innate and adaptive immune responses via inhibition of glycogen synthase kinase-3 (Beurel et al. 2010; Beurel and Jope 2014).

With regard to other psychotropic medications, there were two cases from our series (# 3 and # 7) who had abnormalities of perfusion seen on SPECT and have proven responsive to clozapine. Interestingly, Ertugrul et al. (2009) found that clozapine treatment can increase frontal perfusion in patients studied with SPECT and that the increase is particularly evident in treatment responders.

### Prospect

Given their potential role, circulating thyroid antibodies, whose methods of detection have currently become routinary, should be searched in patients with bipolar spectrum disorders.

With regard to other procedures, none appears currently to be specifically indicated because they await confirmation.

We suggest that future studies investigate in particular abnormalities in perfusion: for example, if there are correlations between specifically involved areas and clinical presentation, outcome, and effect of treatments. Relevance of thyroid antibodies with neuroimaging abnormalities frequently observed in bipolar spectrum disorders, such as white matter hyperintensities in MRI (Gunde et al. 2011), should also be investigated.

Similarly, the role of other antibodies that have been reported to concur with thyroid antibodies is to be confirmed. For example, the search for antibodies against the amino terminal region of alpha-enolase, whose presence have been associated in particular with psychiatric presentation of Hashimoto’s encephalopathy (Yoneda et al. 2007), and AbNMDAR should hopefully become part of routine procedures.

The following observations from this series are to be confirmed and clarified: the concurrence of thyroiditis and mood disorder with persistent tremors and with other autoimmune disorders (diabetes, coeliac disorder, multiple sclerosis, Sjögren syndrome), which is, however, a common finding (Sardu et al. 2012).

## Conclusions

As mood disorders and circulating thyroid antibodies are very prevalent in the population, their concomitant occurrence may be due to chance. Prevalence studies in clinical samples and in community studies are inconclusive. However, based on family and twin studies and on case reports, a role of thyroid antibodies in bipolar spectrum disorders cannot be disregarded.
